# Development of an urban molecular xenomonitoring system for lymphatic filariasis in the Recife Metropolitan Region, Brazil

**DOI:** 10.1371/journal.pntd.0006816

**Published:** 2018-10-16

**Authors:** Anita Ramesh, Mary Cameron, Kirstin Spence, Remy Hoek Spaans, Maria A. V. Melo-Santos, Marcelo H. S. Paiva, Duschinka R. D. Guedes, Rosangela M. R. Barbosa, Claudia M. F. Oliveira, André Sá, Claire L. Jeffries, Priscila M. S. Castanha, Paula A. S. Oliveira, Thomas Walker, Neal Alexander, Cynthia Braga

**Affiliations:** 1 Department of Parasitology, Institute Aggeu Magalhães(IAM/FIOCRUZ Pernambuco), Recife, Brazil; 2 Department of Infectious Disease Epidemiology, London School of Hygiene & Tropical Medicine (LSHTM), London, United Kingdom; 3 Department of Disease Control, LSHTM, London, United Kingdom; 4 Department of Entomology, IAM/FIOCRUZ-PE, Recife, Brazil; 5 Center for Geoprocessing and Statistics, IAM/FIOCRUZ-PE, Recife, Brazil; 6 Department of Virology, IAM/FIOCRUZ-PE, Recife, Brazil; 7 Biological Science Institute, Faculty of Medical Science, University of Pernambuco, Recife, Brazil; University of Georgia, UNITED STATES

## Abstract

**Introduction:**

Molecular xenomonitoring (MX)—pathogen detection in the mosquito rather than human—is a promising tool for lymphatic filariasis (LF) surveillance. In the Recife Metropolitan Region (RMR), the last LF focus in Brazil, *Culex quinquefasciatus* mosquitoes have been implicated in transmitting *Wuchereria bancrofti* parasites. This paper presents findings on the ideal mosquito collection method, mosquito dispersion, *W*. *bancrofti* infection in mosquitoes and *W*. *bancrofti* antigen in humans to aid MX development.

**Methods:**

Experiments occurred within two densely populated urban areas of Olinda, RMR, in July and August 2015. U.S. Centers for Disease Control and Prevention (CDC) light traps were compared to battery-powered aspirators as collection methods, and mosquito dispersion was measured by mosquito mark release recapture (MMRR). Female *Cx*. *quinquefasciatus* were tested by PCR for *W*. *bancrofti* infection, and study area residents were screened by rapid tests for *W*. *bancrofti* antigen.

**Results:**

Aspirators caught 2.6 times more total *Cx*. *quinquefasciatus*, including 38 times more blood-fed and 5 times more gravid stages, than CDC light traps. They also collected 123 times more *Aedes aegypti*. Of the 9,644 marked mosquitoes released, only ten (0.01%) were recaptured, nine of which were < 50m (34.8m median, 85.4m maximum) from the release point. Of 9,169 unmarked mosquitoes captured in the MMR, 38.3% were unfed, 48.8% blood-fed, 5.5% semi-gravid, and 7.3% gravid. PCR on 182 pools (1,556 mosquitoes) found no evidence of *W*. *bancrofti* infection in *Cx*. *quinquefasciatus*. Rapid tests on 110 of 111 eligible residents were all negative for *W*. *bancrofti* antigen.

**Conclusions:**

Aspirators were more effective than CDC light traps at capturing *Ae*. *aegypti* and all but unfed stages of *Cx*. *quinquefasciatus*. Female *Cx*. *quinquefasciatus* traveled short (< 86m) distances in this urban area. Lack of evidence for *W*. *bancrofti* infection in mosquitoes and antigen in humans in these fine-scale studies does not indicate that LF transmission has ceased in the RMR. A MX surveillance system should consider vector-specific collection methods, mosquito dispersion, and spatial scale but also local context, environmental factors such as sanitation, and host factors such as infection prevalence and treatment history.

## Author summary

Lymphatic filariasis (LF) is a parasitic disease transmitted by mosquitoes, and can cause elephantiasis. It is the world’s leading cause of disability due to infectious diseases, affects over 120 million people globally, and is scheduled for global elimination via mass drug administration (MDA) and mosquito control. Molecular xenomonitoring (MX) is a process of screening mosquitoes—not humans—for parasites to estimate whether they are circulating in human populations. MX is especially useful during and following MDA, when new case detection becomes difficult, but is challenging to design and conduct in cities. Using two study sites in the Recife Metropolitan Region, Brazil, we investigated two crucial questions for urban MX development—“What is the best operationally feasible tool to catch adult mosquitoes?” and “How far do mosquitoes disperse in cities?”—in order to determine placement of future surveillance sites. We also screened a proportion of mosquitoes and all eligible residents from the study sites for LF infection. We determined that handheld battery powered aspirators were the best mosquito collection tool; that mosquitoes flew no more than about 85m; and—in this small sample of mosquitoes and very small sample of humans—there was no evidence of LF infection in mosquitoes or study area residents.

## Introduction

Lymphatic filariasis (LF) is a neglected tropical disease and ranked by the World Health Organization (WHO) as the world’s leading cause of physical disability, the second leading cause of long-term disability overall, and the leading cause of disability due to infectious disease [1, 2]. In 2000, an estimated 120 million people were infected with LF parasites and 1.3 billion were considered at risk [3, 4].

The nematode *Wuchereria bancrofti* is responsible for nearly 90% of global LF infections [5]. The mosquito *Culex quinquefasciatus* is the most common vector of urban, nocturnally periodic *W*. *bancrofti* and is thought to be the sole vector of LF in Brazil [6]. In 2000, the Global Program to Eliminate Lymphatic Filariasis (GPELF) aimed to eliminate LF by the year 2020 by interrupting transmission via mass drug administration (MDA) and integrated vector management (IVM) [7]. Since then, 10 GPELF member countries have eliminated LF; of the 73 remaining, 25% are in the ‘surveillance’ phase and 60% have commenced MDA and IVM [8].

LF was introduced to Brazil in the 19^th^ century, and in 1952 the first national LF survey found transmission in 11 states [9, 10]. By the 1990s, after sustained control efforts throughout Brazil, LF remained in three cities in three states: Belém (Pará), Maceió (Alagoas) and Recife (Pernambuco) [9]. In 2018, only Recife and its surroundings remain a focus [9].

The Recife Metropolitan Region (RMR) has a population of over 3.7 million people in 15 municipalities, including Recife proper and the neighboring city of Olinda [11]. In the RMR, nearly 25% of residents live in *favelas* (slums) and areas of suboptimal municipal infrastructure, including proximate to many polluted water bodies (e.g., canals with open sewage) that can serve as *Cx*. *quinquefasciatus* breeding sites [12].

In 2000, overall LF prevalence by thick blood smear (TBS) was 1.34% in Recife and Olinda [13–15]. Recife began MDA in 2005 and Olinda in 2006, with the highest priority areas receiving 5–6 rounds of MDA through 2012. Both cities have assessed transmission by surveying children aged 6–7 years with immunochromatographic card tests (ICTs) to detect circulating filarial antigen (CFA) [16].

Molecular xenomonitoring (MX) is the use of molecular methods, such as polymerase chain reaction (PCR), to detect pathogen DNA or RNA in the vector as a proxy for infection in the human population. MX is a promising method for monitoring LF transmission, MDA and IVM success, and LF elimination [17–21]. Over nearly twenty years, MX has been tested in a variety of LF-endemic settings with different vector-parasite dynamics, with evidence of its utility from five out of six WHO Regions: Africa/AMRO (Ghana, Sierra Leone, Tanzania), Americas/AMRO/PAHO (Trinidad and Tobago), Eastern Mediterranean/EMRO (Egypt), Southeast Asia/SEARO (India, Sri Lanka), and Western Pacific/WPRO (American Samoa, French Polynesia, Samoa) [21–30].

MX could prove more appropriate and useful as control activities reduce LF transmission, because after MDA parasitological detection tools such as TBS become less sensitive while immunological detection tools such as ICTs become less specific [31]. Moreover, it would be necessary to screen large population samples to detect low and clustered transmission areas.

For MX, it is crucial to capture adult female mosquitoes so that they can be screened for infection (any parasitic stage) as well as infectivity (the L3 larval stage), the latter being the most precise determinant of transmission potential. Several MX protocols have been developed for *Cx*. *quinquefasciatus* [21, 32–35].

Currently, there is no universally recommended strategy for MX sampling or tool for MX collection and MX programs differ by site-specific vector and parasite dynamics. In Tanzania, for example, U.S. Centers for Disease Control and Prevention (CDC) gravid traps collected the greatest number of *Cx*. *quinquefasciatus* of all stages as well as gravid stages in relation to four methods. However, a subsequent comparison of CDC light vs. CDC gravid traps demonstrated that both caught similar numbers of mosquitoes, although of different gonotrophic status, and that CDC light traps collected more infected mosquitoes [24]. In the RMR, the preferred method for adult mosquito collection is aspiration, which also has the benefit of preferentially collecting post-blood meal, resting mosquitoes. This is advantageous for LF elimination and pathogen surveillance because blood-fed, gravid, and semi-gravid mosquitoes are more likely to have ingested mf-infected blood.

To date, a collection method comparison (CMC) for *Cx*. *quinquefasciatus* including aspiration has not been published from any urban setting. Therefore, there is a dearth of evidence on collection tools, such as aspirators, and program-oriented techniques for *Cx*. *quinquefasciatus* mosquitoes, which dominate in urban areas [19]. For instance, in the RMR, fixed battery-operated or energy-source requiring traps (e.g., BG sentinel, CDC light) are discouraged due to battery theft and power cuts, whereas trapping methods that rely on attractants (e.g., gravid, sticky ovitraps) cannot be placed inside domestic spaces due to residents’ distaste for the strong odors emitted. A CMC including aspiration in this setting would aid LF elimination and MX system development planning.

Ideally, MX methodology should take into account the geographical scale and directionality of mosquito dispersion as the former is related to the spatial scale of disease transmission [36, 37]. Understanding mosquito dispersion in a given setting allows public health officials to more accurately plan the limits of where related vector borne disease may occur, and thus where control efforts should concentrate. Although some studies have included evaluations of different mosquito collection methods, none have formally assessed mosquito flight distance and patterns within the context of MX and none has occurred in a densely populated, urban area [21, 24, 28, 30, 38–40].

One of the most straightforward methods for measuring mosquito dispersion is mosquito mark-release-recapture (MMRR), but most MMRR studies of *Cx*. *quinquefasciatus* have been conducted in high-income countries (e.g., United States) and among rural settings (e.g., dairy farms) [36, 41–44]. While rural MMRR studies indicate that *Cx*. *quinquefasciatus* can travel up to 2 km for host blood seeking and oviposition, the only published ‘urban’ *Cx*. *quinquefasciatus* MMRR study occurred in a central Texas university town of approximately one eighth the population density of RMR *favelas* [45]. For comparison, studies on *Ae*. *aegypti* dispersal, including in urban areas of Brazil (often set within less population dense / forested areas of cities), indicate that *Ae*. *aegypti* tend to fly 100m or less [46, 47].

Despite the promise of MX for LF and other vector-borne pathogens, there are no published reports of its use to detect *W*. *bancrofti* infection in *Cx*. *quinquefasciatus* in urban settings. CMC studies could provide information on effective, practical, and acceptable collection tools for MX programs. MMRR studies could provide crucial insights on LF risk and transmission, especially if mosquito parameters (e.g., mean distance travelled, MDT) are combined with those on human infection over the same space and, ideally, time. As LF elimination efforts continue, and eventually are localized to difficult-to-treat urban areas, information on mosquito dispersion in such settings is of increasing importance.

The following strategies were employed to develop a MX system in the RMR: (i) CMC to determine whether battery-powered aspirators or CDC light traps more efficiently collect *Cx*. *quinquefasciatus* females; (ii) MMRR to estimate mosquito dispersion to determine grid size for use in subsequent surveillance; (iii) molecular screening via PCR in a sample of female *Cx*. *quinquefasciatus* to determine *W*. *bancrofti* infection in mosquitoes; and (iv) immunological screening via ICT to detect *W*. *bancrofti* antigen in study area residents.

## Methods

### Study site characteristics

**Fig 1:**
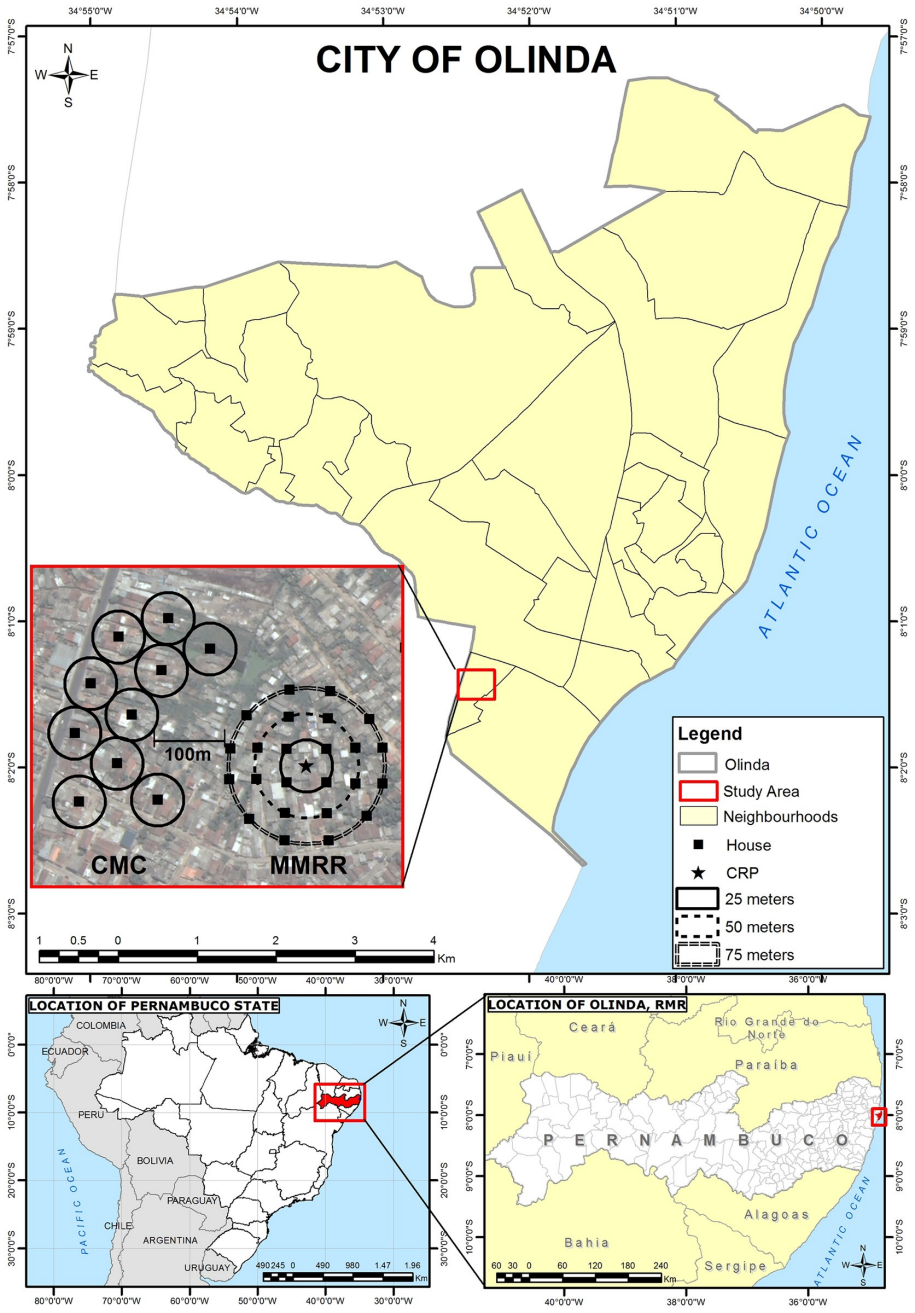
Map of city of Olinda, Recife Metropolitan Region (RMR), Pernambuco State, Northeastern Brazil, and planned study sites within Sítio Novo: Collection method comparison (CMC); mosquito mark release recapture (MMRR); 100m buffer zone.

The CMC, MMRR, molecular screening of mosquitoes, and immunological screening of study residents were conducted in two selected areas within the neighborhood of Sítio Novo in the city of Olinda, RMR. Olinda is the second most populous and population-dense city of the RMR, with 377,779 residents in its area of 41.68 km^2^ (Fig 1) [11]. It has a tropical monsoon climate (Köppen climate classification = *As*), and temperatures range from 30 °C (86 °F) in January and February to 21 °C (70 °F) in July [48]. Peak dry season is in November (average 36mm rainfall) while the rainy season, extending June—August, peaks in July (average 388 mm rainfall) [49, 50]. Data collection occurred between July 22 and August 21, 2015, coinciding with the end of rainy season and associated peak in mosquito abundance.

### House selection

Houses were selected using satellite images and geographic information systems (GIS) software of ArcGIS 10.2 (ESRI 2014. ArcGIS Desktop: Release 10. Redlands, CA: Environmental Systems Research Institute) and QGIS 2.10.1(QGIS Development Team (2015). QGIS Geographic Information System. Open Source Geospatial Foundation Project. http://qgis.org). House selection accounted for geographic (aligning along transport arteries in CMC) and environmental (e.g., avoiding mangrove in MMRR) barriers, as well as local health authority advice on the most secure areas to work. In the field, study teams used a combination of global positioning system (GPS) devices (Garmin GPSmap 76cs, 3m precision) and GIS / satellite image maps to locate selected houses. If residents were not willing or able to participate, including providing regular access over four weeks, then alternative houses were enrolled by selecting houses to the right, then left, then opposite the initial house until an appropriate alternative could be found. To avoid contamination, the CMC and MMRR study areas were separated by a buffer zone of 100m based on the estimated average mosquito flight distance from urban *Ae*. *aegypti* dispersion studies (Fig 1).

**Fig 2:**
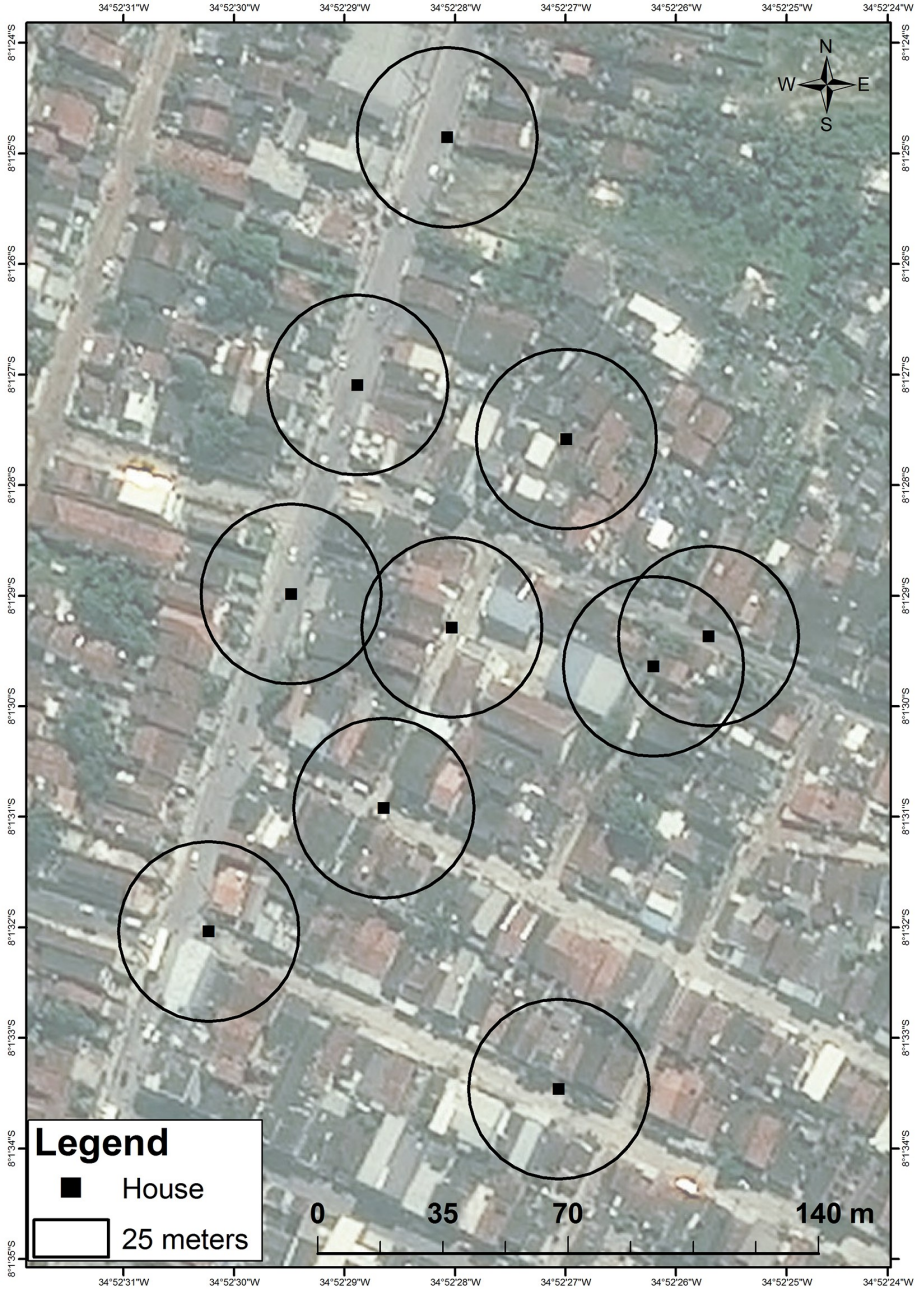
Collection method comparison (CMC) mosquito collection points via handheld aspirators and CDC light traps, Sítio Novo, Olinda, RMR, Brazil, July 22–August 21, 2015.

The CMC occurred in a commercial and residential zone with some paved streets, municipal sanitation, and drainage systems. Houses were of higher quality construction, with brick walls, solid/permanent roof, some partially screened windows, and fewer wall openings, than those in the MMRR area. Still, much of this area was considered to be of suboptimal housing, including favelas. As much as possible, houses were selected along main streets in order to provide better access for equipment transfer (Fig 2).

**Fig 3:**
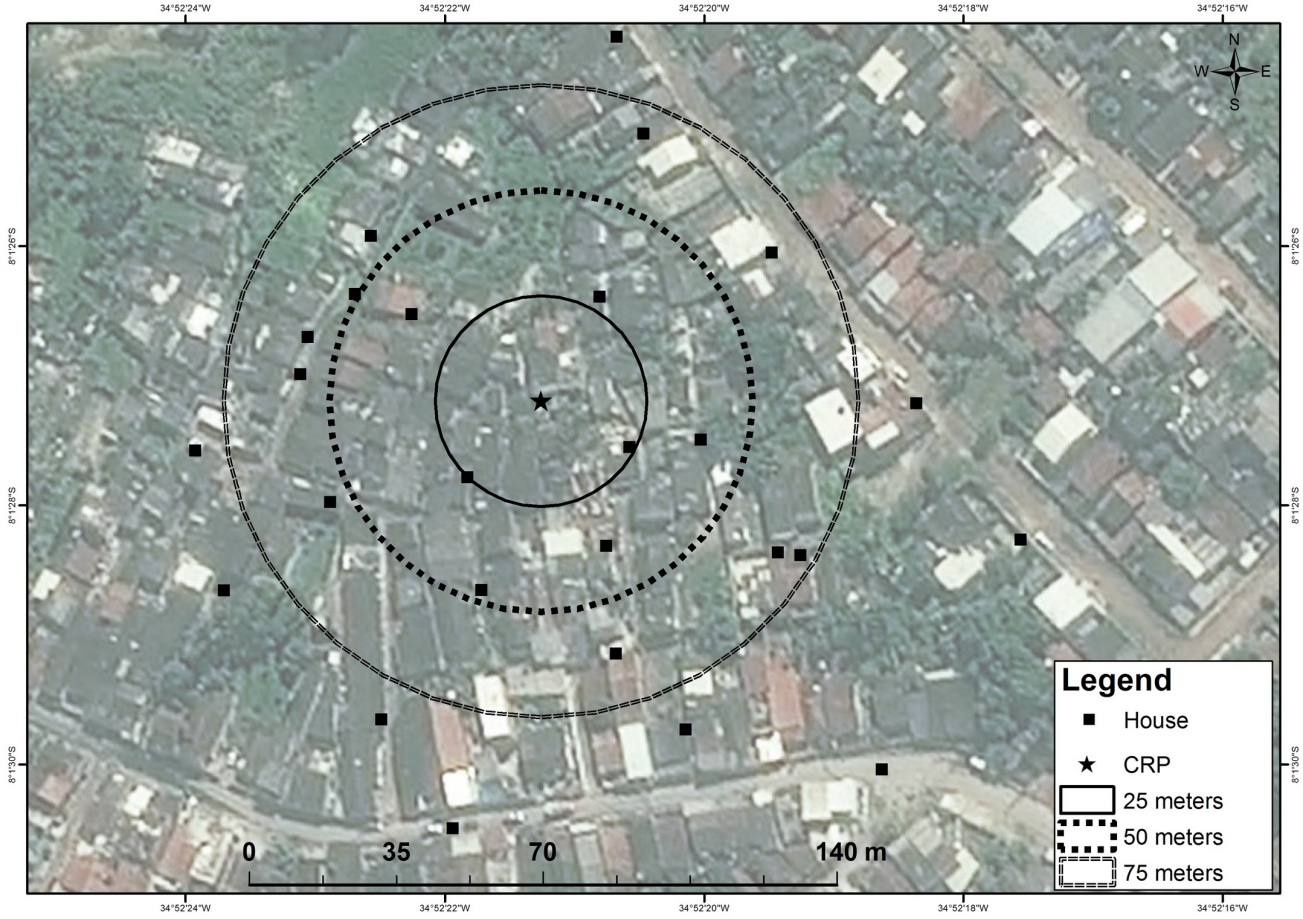
Mosquito mark release recapture (MMRR) collection points via handheld aspirators, Sítio Novo, Olinda, RMR, Brazil, July 22–August 21, 2015.

The MMRR occurred in an infrastructure-lacking residential area with poorly paved streets, sanitation, and drainage. During the study period, houses were often flooded from an adjacent area of riverine mangrove (Fig 3).

Molecular screening of *Cx*. *quinquefasciatus* and immunological screening of study participants occurred in CMC and MMRR study areas, from where the samples for each were obtained.

### Study designs

#### Collection method comparison (CMC)

The CMC study employed a crossover design to compare the ability of battery-powered handheld aspirators and CDC light traps to capture adult female mosquitoes in terms of (a) total numbers of female *Cx*. *quinquefasciatus*, (b) *Cx*. *quinquefasciatus* by physiological status (unfed, blood-fed, semi-gravid, gravid), and (c) total numbers of female *Ae*. *aegypti*.

The sample size calculation was based on a mean and standard deviation of 10.12 ± 5.37 egg rafts, a *proxy* for gravid female mosquitoes collected per trapping night in a single ovitrap (BR-OVT) in the RMR [51]. This indicated that five houses per method (aspiration or CDC light trap) over four nights in each of four weeks (i.e., a total of 80 trapping nights per method) could detect a difference as low as 25% between the means of each collection method with 80% power, or a 30% difference at 90% power, in a 2-tailed test. A total of 10 houses within an area of 300m x 400m were selected (Fig 2).

Each week, the CMC houses were sampled as follows: a) five houses received CDC light traps Monday—Thursday night with nets collected the following morning; and b) five received aspirators over the same four days when CDC nets were collected (Tuesday—Friday mornings). These four collection days per week, over four weeks, provided 16 trapping days per treatment arm and, since there were 5 houses receiving treatment each day, resulted in the calculated sample size of 80 trapping nights per collection method. Due to logistical constraints, traps could not be hung on Sunday nights, nor could batteries be changed on Monday mornings. The CMC was designed to maintain a study buffer of 50m between each of the 10 participating houses, but geographical constraints such as major roads, and instruction from the local health authorities, prevented this from being uniformly implemented. Ultimately, two houses were located more than 100m from adjacent houses and another two houses were located within 50m of each other (Fig 2).

#### Mosquito mark release recapture (MMRR)

The MMRR study involved the following stages: 1) rearing larvae to adults, 2) mating sterile males with females, 3) fluorescent dust marking of adults, predominantly females, 4) releasing marked adults from a central release point (CRP), and 5) recapturing. Stages 1–3 occurred in the laboratory; stages 4–5 in the field.

#### Rearing

*Cx*. *quinquefasciatus* (CqsLab colony, 45th generation, IAM/FIOCRUZ insectary, Recife, Brazil) larvae were reared in plastic containers and fed with Friskies fish flavored cat food; temperatures of 24.4°C—27.6°C and humidity of 63%–80% were maintained. Upon emergence, adult mosquitoes were held in wire mesh cages and fed on a 10% glucose solution; temperatures of 24.9–26.9°C and humidity of 66%–81% humidity were maintained.

#### Mosquito sterilization and mating

Pre-field pilot testing indicated deviances from expected survival and flight patterns of marked female *Cx*. *quinquefasciatus*. Thus, in order to avoid any resultant changes in flight behavior while still reducing the potential of disease transmission from liberating female mosquitoes, female *Cx*. *quinquefasciatus* were mated with irradiated males, so that their spermathecae would contain sterile sperm. Within 24–48 hours after passing through the larval stage, male pupae were irradiated with 40 Gy; adult males emerged approximately one day after radiation. Mating of irradiated males with untreated females occurred one day before the marking procedure. Mating effectiveness, measured by the number of female mosquitoes that oviposited after mating, was determined to be 50–60%.

#### Mosquito marking in the laboratory and field

The following fluorescent powders (Sterling Colour, 850 Series, http://www.sterling-colour.co.uk) were used: Red3 for the test release (R1), and Yellow, Magenta, and Red3 for the three experimental releases (R2, R3, R4, respectively). For marking, small batches of adult female *Cx*. *quinquefasciatus* were mouth aspirated into individual, gauze-covered 140ml paper cups (batches of 30 for R1—R3, but batches of 40 for R4) until the required number to be released per round was reached. Cups were placed inside a -20°C freezer for up to 15 minutes to ensure mosquitoes lacked mobility. After removal, a 5ml syringe was filled to 0.5 ml with fluorescent powder, using a 0.7mm x 25mm needle, and the needle was inserted at a 90° angle through the gauze. A dust cloud was created inside the cup by pushing down the syringe. After marking, adult female mosquitoes were fed on a 10% glucose solution and held overnight in wire mesh cages, with gauze netting, at the IAM/FIOCRUZ insectary before release the following day.

#### Release

A satellite image and a pre-programmed GPS device was used to select a central release point (CRP), which was set in the center of a 5m x 5m courtyard that was surrounded by a combined, large family dwelling of four houses. Sentinel houses were selected for aspiration based on their location on radii of approximately 25m, 50m and 100m from the CRP (Fig 3).

**Fig 4:**
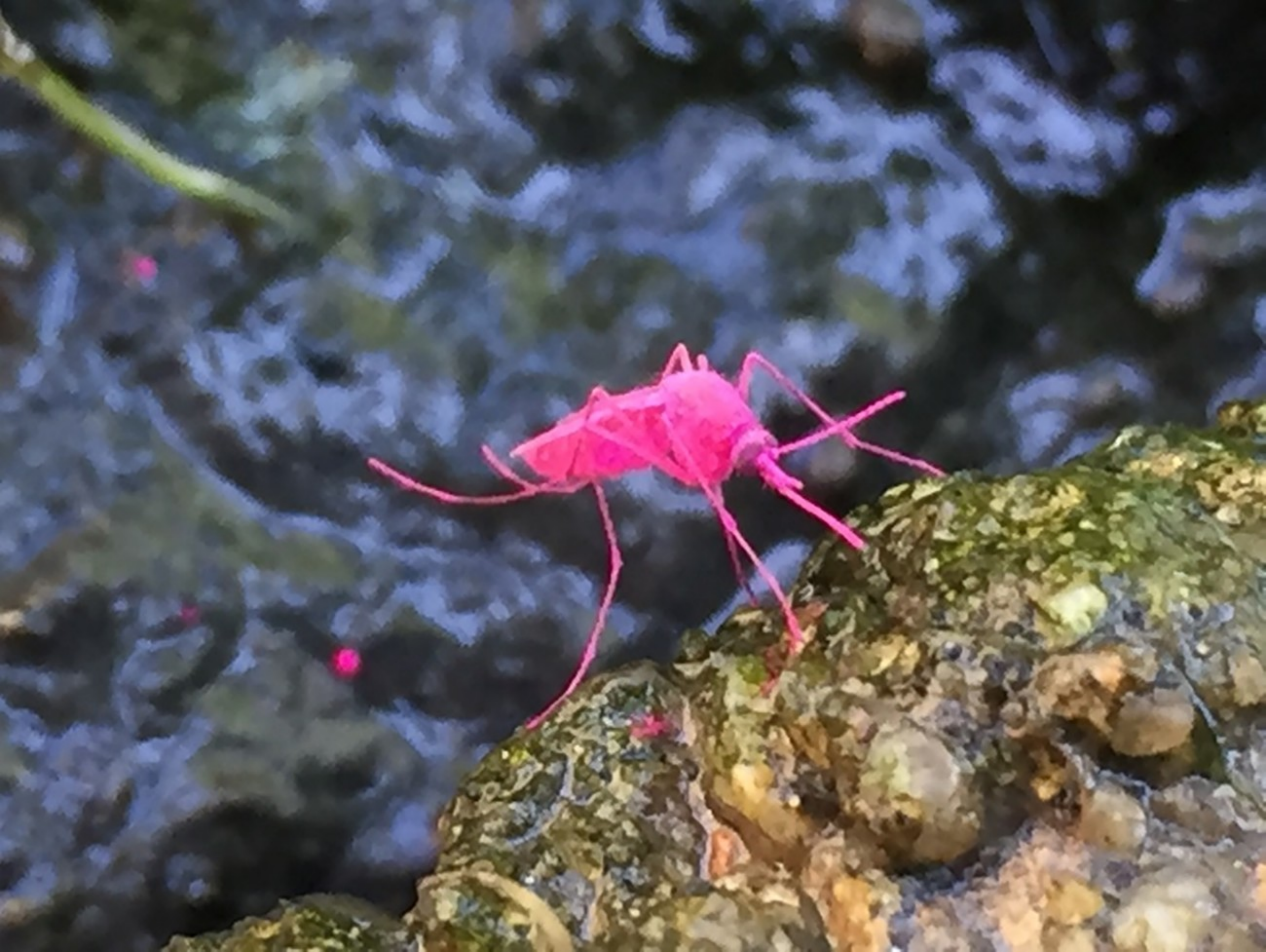
Magenta-marked adult female *Culex quinquefasciatus* in field conditions Sítio Novo, Olinda, RMR, Brazil, July 22–August 21, 2015. [Photo: Anita Ramesh, LSHTM/IAM/FIOCRUZ].

During each release, marked mosquitoes (approximately 1000 mosquitoes/cage) were placed on the ground in three fixed positions within the CRP; cages were opened, and gently tapped over a period of 30 minutes to encourage dispersal (Fig 4). Mosquitoes that did not fly away after 30 minutes (including those that died) were counted in order to quantify how many marked mosquitoes were actually liberated.

#### Recapture

**Fig 5:**
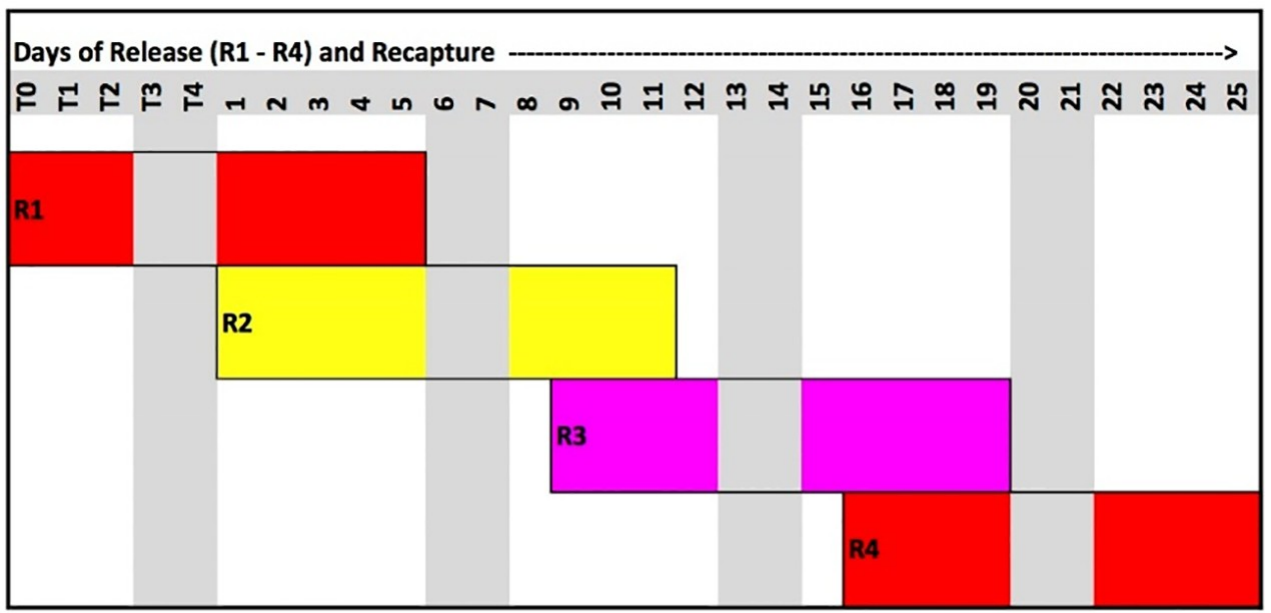
Release and recapture schedule of marked mosquitoes per day of release (R1–4) and recapture, Sítio Novo, Olinda, RMR, Brazil, July 22–August 21, 2015.

Based on available literature indicating significantly diminishing returns beyond a recapture of two weeks from release, a maximum recapture period of 10 working days (five days per week over two weeks) was planned per round [36, 52, 53]. During the experimental period (R2 –R4), three different cohorts of marked mosquitoes were released over three consecutive weeks; with each cohort followed for two weeks (10 working days), this provided an experimental period of four weeks for three rounds of release and recapture (Fig 5).

For recapture, 24 houses were included with distances of radii from the CRP as follows: seven houses within 50m (minimum 24m), nine houses within 50-75m, six houses within 75-100m, and three beyond 100m (maximum 119m). Field teams used battery-powered aspirators, following the same protocol as the CMC study, for two weeks after each release. Aspirators were the method of choice of IVM staff, due to their ease of use and deployment, and acceptability and familiarity among residents. Houses were aspirated daily Monday—Friday over a period of four weeks, for a total of 20 collection days. Field teams were appointed to each of four quadrants per week; teams were rotated clockwise each week to minimize data collection bias.

### Entomological tools and protocols

#### Handheld aspirator

Large, handheld, battery-operated aspirators were used for both the CMC and MMRR studies (Appendix I; http://www.horstarmadilhas.com.br). A collection net was attached at the bottom end of the aspirator and the battery pack was holstered in a belt that secured the wires leading to the fan. Each week, houses were aspirated daily from 9 am—11:30am, considering security, logistics, and that *Cx*. *quinquefasciatus* are more likely to be caught resting post-blood meal on walls in the morning. Each house was aspirated for 15 minutes as standard, but timing allocation depended on type of house: e.g. five minutes in each in the following areas: (i) living room, (ii) bedroom(s) and (iii) internal toilet or external toilet/septic tank/ water storage tank/water distribution box.

#### CDC light trap

The CDC light traps used in the CMC (Appendix I; http://www.horstarmadilhas.com.br) consisted of a trap with a light source, and battery-powered fan, and a collection net to retain mosquitoes. Study teams hung traps at heights between 1.5–2m from the floor in bedrooms of participating houses; traps were hung via hooks and nails that were already affixed to the walls. Study teams demonstrated how to turn on the CDC light traps to the head of household (HoH), and requested they do so each night at 6pm (sunset). Study teams returned the following morning (Tuesday—Friday) between 9:30–11:30am to collect the nets of trapped mosquitoes as well as replace collection nets and batteries for use that evening.

### Post-collection mosquito storage, transport, and processing

Mosquito collection nets were placed in an open-top storage box and transported back to the IAM/FIOCRUZ Insectary within two hours of field collection. Upon arrival, nets were immediately placed in a -20°C freezer for at least 20 minutes to immobilize the mosquitoes. Mosquitoes were then removed from the freezer and placed on ice for identification, sex determination, and assessment of female physiological status. The numbers and status of female *Cx*. *quinquefasciatus* mosquitoes were recorded per house, per day. Female *Cx*. *quinquefasciatus* and *Ae*. *aegypti* mosquitoes were placed in Eppendorf tubes (maximum of 50 per tube, separated by species) labeled per house per day and stored in a -80°C freezer for future analysis.

### Contextual data

Meteorological data (temperature, humidity, wind) that could influence mosquito flight range, survival and dispersal were obtained from the Brazilian National Meteorological Institute (INMET: www.inmet.gov.br) and the Pernambuco State Agency for Water and Climate (APAC: www.apac.pe.gov.br) [49, 50].

### Molecular screening of *Cx*. *quinquefasciatus* for *W*. *bancrofti* infection

Female *Cx*. *quinquefasciatus* mosquitoes were pooled into groups of up to 10 per pool depending on study area. Pooling was done by house per day (MMRR) or by house per week (CMC). RNA was extracted using a Ambion Trizol-based protocol (see appendix III) and RNA was re-suspended in 30 μl of Invitrogen Ultrapure water and stored at -80°C to preserve RNA prior to reverse transcription. RNA samples were reverse transcribed using a QIAGEN QuantiTect reverse transcription kit according to manufacturer’s instructions. Successful generation of cDNA was confirmed by real time PCR assays targeting the *Cx*. *quinquefasciatus S7 mRNA* gene using QIAGEN QuantiTect Sybr Green Master Mix. *W*. *bancrofti* detection was undertaken using a Taqman real time PCR assay targeting the constitutively expressed *tph-1* gene using Promega GoTaq Probe qPCR Master Mix [54]. See Appendix III for more details.

### Immunological screening of study areas residents for *W*. *bancrofti* antigen

Concurrent to CMC and MMRR activities, immunochromatographic card tests (AD12-ICT card test, NOW Filariasis) were requested from all eligible (age 2–65 years) and consenting residents of the 35 houses in this study. This test detects CFA using the monoclonal antibody AD12, which recognizes a 200-kDa filarial antigen from either adult worms or microfilariae. [55] Study teams approached each HoH and any available household members, presented an information sheet and an invitation to receive ICT screening at the local community center. Any resident who did not attend the community center was visited at least three times to attempt to administer the ICT in their residence. The test was performed according to the manufacturer’s instructions and read by trained technicians in the field after 10 minutes. Visualization of two lines (test and control) was interpreted as a positive result.

### Data analysis

Data were double entered by two independent data entry staff, cleaned, and analyzed with Stata 14 (StataCorp. 2015. *Stata Statistical Software*: *Release 14*. College Station, TX: StataCorp LP).

#### CMC study

The mean numbers of adult female mosquitoes by species and physiological status were compared between the two capture methods. As data were overdispersed, a negative binomial regression was used to detect differences between treatments. For *Culex*, house and night were included in the model, as additional independent variables, as random and fixed effects, respectively, the former with a beta distribution. This was not possible for *Aedes* because of small numbers in the CDC traps. Data were excluded from analysis in instances of battery failure, premature disengagement of the CDC light traps (e.g., participants turning them off), and inability to enter participants’ houses on any given day for mosquito collection.

#### MMRR study

Results from pilot Test Release 1 (R1) were excluded from analysis. Statistical analysis was performed using Stata 14, with straight line distances calculated from the CRP using ArcGIS 10.2 in order to establish MDT and maximum distance travelled, as well as mean wind direction using circular statistics [56].

### Informed consent, confidentiality, and ethical approval

Study aims and methods were presented to HoHs and verbal and written informed consent was sought; households were enrolled upon receipt of written informed consent. All names, addresses, and GPS coordinates of participating HoHs and residents were concealed from study staff apart from the principal investigator and study coordinator, both of whom held the linking keys. Field teams worked during the mornings of weekdays due to security concerns as well as to increase acceptability of daily aspiration or CDC light trap placement/net collection. Ethical approval was obtained from the Research Ethics Committees of the Instituto Aggeu Magalhães (IAM/FIOCRUZ) and the London School of Hygiene & Tropical Medicine (LSHTM) prior to the commencement of fieldwork. [CAAE: 44535515.0.0000.5190; LSHTM: 10276; 10185].

## Results

### CMC study

Of a total of 80 trapping nights planned, 78 were obtained for battery-powered aspiration, and 68 for CDC light traps. The primary reasons for losses in trapping night measurements were battery failure (especially for CDC light traps left overnight) and inability to enter participants’ houses.

Table 1 presents the number of mosquitoes collected by collection method, species and physiological status. A total of 970 adult females of *Cx*. *quinquefasciatus* (unfed = 393, blood-fed = 403, semi-gravid = 165, gravid = 9) were captured, of which 684 were by aspiration and 286 by CDC light traps. A total of 188 *Ae*. *aegypti* were captured, all but one by aspiration.

**Table 1:** CMC study: Number of female *Cx*. *quinquefasciatus* and *Ae*. *aegypti* mosquitoes collected, with physiological status (*Cx*. *quinquefasciatus*), incidence rate ratio (IRR), and models for *Cx*. *quinquefasciatus* including collection method and adjusted by house, day, and week, Sítio Novo, Olinda, RMR, Brazil, July 22–August 21, 2015. Each pair of rows is a separate analysis.

Mosquitoes by Collection Method and Physiological Status		N	IRR (95% CI)	Z	P value
***Cx*. *quinquefasciatus***					
Total Females	CDC light trap	286	1.00	-	-
	Aspirator	684	2.64 (1.99, 3.51)	6.73	<0.001
Unfed	CDC light trap	251	1.00	-	-
	Aspirator	142	0.74 (0.53, 1.04)	-1.76	0.079
Blood-fed	CDC light trap	9	1.00	-	
	Aspirator	394	38.4 (18.6, 79.1)	9.89	<0.001
Semi-gravid^a^ & gravid	CDC light trap	26	1.00	-	-
	Aspirator	148	5.79 (3.59, 9.34)	7.21	<0.001
***Ae*. *aegypti***					
Total Females	CDC light trap	1	1.0	-	-
	Aspirator	187	122.6 (25.1, 903)	4.72	<0.001

^a^Only 9 semi-gravid mosquitoes were caught, all by aspiration.

Adjusting for the house and night factors, aspirators caught 2.6 times more total females, and 38 times more blood-fed mosquitoes than CDC light traps (all p<0.0001). Aspirators caught 5.8 times more gravid and semi-gravid mosquitoes than CDC light traps; these abdominal conditions were pooled due to the small number (nine) of semi-gravid mosquitoes collected. Aspirators collected almost 25% fewer unfed (p<0.0001) *Cx*. *quinquefasciatus* than CDC light traps. Aspirators collected 123 times more (p< 0.0001) total females of *Ae*. *aegypti*.

### MMRR study

Data from the experimental period (R2 –R4) were collected over a period of 19 days in 25 households, for a total number of 475 observations. The study recruited 24 houses as planned, but one house refused access to the field team after the first week, so another was recruited in its place to preserve measurements across a theoretical grid (the size of which would be measured along radii emanating from the CRP). This newly recruited 25^th^ house then participated for 3 weeks, yielding four weeks of collections from each of 23 houses, one week from the first house that dropped out, and the remaining three weeks from the 25^th^ house.

#### Release, recapture, and MDT of marked mosquitoes

Table 2 illustrates the recapture rate and mean distance travelled (MDT). A total of 10,163 marked adult mosquitoes (7,614 female, 2,549 male) were released from R1-R4. During the experimental period, a total of ten (0.01%) live, marked mosquitoes were recaptured, with five mosquitoes recaptured from each of two rounds (R2 and R4). Four out of five mosquitoes from R2 were recaptured in the quadrant south-east of the CRP. Three days after the second release two mosquitoes were recaptured at similar angles from the CRP. All five mosquitoes from the last release (R4) were recaptured within the same house, located south-west of the CRP. The MDT was 34.8m, and the furthest recapture point from the CRP was 85.4m; nine out of ten mosquitoes (90%) were found within 50m of the CRP.

**Table 2:** MMRR study: Number of *Cx*. *quinquefasciatus* mosquitoes released, number and proportion recaptured, and mean distance travelled (MDT), Sítio Novo, Olinda, RMR, Brazil, July 22–August 21, 2015.

Release color and number (R1-4)		Number females (F) and males (M) released		Number recap-tured	Proportion recap-tured	Mean distance (meters) travelled by day (D) after release			
						D1	D2	D3	D4
-	R1	F	379	0	0.0000	-	-	-	-
		M	140	0	0.0000	-	-	-	-
						-	-	-	-
-	R2	F	3388	3	0.0009	-	-	61.6	37.1
		M	770	2	0.0026	-	-	23.7	37.8
						-	-		
-	R3	F	2041	0	0.0000	-	-	-	-
		M	1010	0	0.0000	-	-	-	-
						-	-	-	-
-	R4	F	1806	5	0.0028	25.2	25.2	25.2	-
		M	629	0	0.0000	-	-	-	-

#### Unmarked mosquito capture

In addition to the marked mosquitoes collected as the main mosquitoes of interest to determine dispersion, the majority of mosquitoes collected via aspirators were unmarked. A total of 18,286 unmarked *Cx*. *quinquefasciatus* mosquitoes were captured over 18 collection days; of these, 9,169 (50.1%) were female. Among females, 3,512 (38.3%) were unfed, 4,478 (48.8%) blood-fed, 507 (5.5%) semi-gravid and 672 (7.3%) gravid. A total number of 1,444 *Ae*. *aegypti* mosquitoes were captured, of which 722 (50%) were female.

#### Climate

The mean wind direction over all collection days (R1-4) was 161.0° (95% CI: 157.3–164.6), coming from a South-South-Eastern direction. The average wind speed was 1.65m/s (95% CI: 1.57–1.72), with reported wind gusts between 0.7 and 11.9m/s. Temperature was relatively stable over the study period, with an overall mean of 24°C (95% CI: 23.80–24.16). Rainfall varied considerably over the study period, especially during the first week of the experimental period (R2). It did not rain on 73.19% of the total 25-day period, but, on those days it rained, the median rainfall was 6.9mm (IQR: 0.4–12.8). Humidity was relatively stable, with a median of 0.79 (IQR 0.66–0.86).

### Combined results from CMC and MMRR collections

In total, the CMC and MMRR experiments collected 10,139 (970 CMC, 9169 MMRR) *Cx*. *quinquefasciatus* and 910 (188 CMC, 722 MMRR) *Ae*. *aegypti* female mosquitoes (Table 3). All *Ae*. *aegypti* and *Cx*. *quinquefasciatus* mosquitoes were stored at -80C to preserve RNA (filarial, arboviral) for future analysis; a subset (15% of the total yield) of *Cx*. *quinquefasciatus* was then subjected to molecular analysis.

**Table 3:** Total numbers of female *Cx*. *quinquefasciatus* and *Ae*. *aegypti* mosquitoes captured in CMC and MMRR experiments, Sítio Novo, Olinda, RMR, Brazil, July 22–August 21, 2015.

Study Area	*Cx*. *quinquefasciatus*					*Ae*. *aegypti*
	*Unfed*	*Blood-fed*	*Semi-gravid*	*Gravid*	*Total*	*Total*
CMC	393	403	9	165	970	188
MMRR	3512	4478	507	672	9169	722
**Total**	*3905*	*4881*	*516*	*837*	*10139*	*910*

### Molecular screening of *Cx*. *quinquefasciatus* for *W*. *bancrofti* infection

Of the 10,139 *Cx*. *quinquefasciatus* collected (Table 3), 182 pools (112 CMC, 70 MMRR) representing 1,556 (856 CMC, 700 MMRR) female mosquitoes of all abdominal conditions were screened for *W*. *bancrofti* infection. PCR analysis confirmed successful generation of *Cx*. *quinquefasciatus* cDNA from each mosquito pool but revealed no evidence of *W*. *bancrofti* infection.

### Immunological screening of study areas residents for *W*. *bancrofti* antigen

A total of 110 (99.1%) out of a reported 111 full and part-time residents of the 35 houses included in the CMC and MMRR studies underwent immunological analysis via ICT. The majority were female (63%), and the gender disparity was more evident in older age groups. Nearly 25% of the population undergoing ICT was 61 years of age or older. None tested positive for *W*. *bancrofti* CFA.

## Discussion

MX is a promising method to monitor LF transmission, especially during the ‘endgame’ of LF elimination. The two experiments presented in this paper inform the development of a gridded MX system in a densely populated urban area where a single parasite (*W*. *bancrofti*) is likely transmitted by a single vector (*Cx*. *quinquefasciatus*). This contrasts with the majority of MX-related studies to date, which have been conducted in rural areas where more than one vector and / or parasite may be implicated in LF transmission.

### CMC study

This study compared battery-powered handheld aspirators with CDC light traps, although several other methods had been considered. Gravid traps were rejected due to acceptability concerns related to the smell of attractants (e.g., grass infusion) if used indoors, logistical issues related to transporting large volumes of infusions, and trap placement in relation to security (e.g. theft) given extremely limited secure outdoor space for each house in the study. A paper by Irish et. al. found that the gravid traps caught less infected *Cx*. *quinquefasciatus* mosquitoes than CDC light traps [24]. BG sentinel traps were rejected due to their large size, unwieldiness and fan noise. Sticky ovitraps were rejected based on local expert advice and experience that they are vastly inferior to battery-powered aspiration, and genetic material (RNA) in collected mosquitoes would likely be degraded due to desiccation.

The nearly three-fold superiority of aspiration in collecting female *Cx*. *quinquefasciatus* may be surprising, given that many other sites preferentially use CDC light or gravid traps for this species [24, 26, 27, 38, 40, 57, 58]. However, much of the existing literature is based upon studies conducted in rural settings with different residential and sanitation infrastructure and low population density. One previous study in the metropolitan area of Recife found that CDC light traps collected an average of 55 *Cx*. *quinquefasciatus* females per trap in 1991–2 [59]. While this quantity is much greater than that found in the current study, one possible explanation of this result is an improvement of infrastructure and sanitation within Olinda over the past two decades.

Aspirators collected 25% fewer unfed *Cx*. *quinquefasciatus* than CDC light traps, consistent with CDC light traps preferentially attracting pre-blood meal and aspirators collecting post-blood meal mosquitoes [24]. Furthermore, aspirators collect mosquitoes resting indoors, which are less likely to be unfed females [60]. As female *Cx*. *quinquefasciatus* mosquitoes are endophagic and endophilic, battery-powered aspiration inside houses should be more likely to collect resting blood-fed females. This was the case in the Sítio Novo, where aspirators collected 47 times more blood-fed *Cx*. *quinquefasciatus* than CDC light traps. CDC light traps collected less than 2% of the blood-fed females, which is much lower than most previous studies [30, 40, 60], although in line with one recent study in Tanzania [24].

Several other limitations should be noted. First, there were more operational issues surrounding the use of CDC light traps than aspirators. Light and noise emitted from the traps were aggravating to several residents; three participating households requested the CDC light traps be removed from their bedrooms at night. Of trapping nights lost, nine were due to battery failure, two were lost due to traps being prematurely switched off, and one was due to participants not being at home. By contrast, only two data points were lost during aspiration, both due to participants not being home during morning visits. This also raised another important issue. As CDC light traps require a freshly charged battery each night, if a house cannot be accessed each morning, then the previous night’s collection net cannot be retrieved and a new battery cannot be swapped. This effectively means that not being able to access a house during CDC light trap testing results in losing two nights’ of trapping data, whereas not being able to access a house for aspiration results in only one data point being lost.

Of the 188 *Ae*. *aegypti* captured, 99% were collected by aspirators; so, unlike CDC light traps, they may also offer an alternative tool to sticky ovitraps for collecting adult female *Ae*. *aegypti* [30, 40, 61–67]. The finding that aspirators collect adult *Ae*. *aegypti* extremely well, and the possible co-circulation of arboviruses with LF, indicate that a combined MX and surveillance program for several vector-borne diseases could be both time- and cost-effective [68].

Aspiration of resting mosquitoes is not a new collection method for vectors of LF, and has already been successfully adopted for xenomonitoring surveillance during and after MDA programs in Egypt and India [69, 70]. However, normally these studies involved other collection methods (e.g., CDC light traps), and not the large, battery-powered aspirators used in the current study. The type of aspirator used here provides a promising tool for a xenomonitoring program for the RMR. While aspiration has for some time been the locally preferred method of collecting adult resting mosquitoes by Secretary of Health officials, no standard operating procedures have previously been in place. The present study produced an easy to use written protocol that local researchers (including those in other research groups) are currently using in order to standardize adult vector collection.

Since aspiration tends to collect a significantly higher proportion of blood-fed mosquitoes than some other methods, any PCR-positive samples could, in principle, be attributed to the mosquito having recently ingested an infected blood meal, as opposed to carrying an established infection. In an MX program, this could potentially inflate the infection rate beyond the true transmission potential [71]. It has likewise been argued that other collection methods that preferentially capture older and previously blood-fed mosquitoes, such as gravid traps, would have a higher likelihood of detecting infective L3 larvae [38]. The introduction of a reverse transcriptase based PCR assay however, would negate the need to exclude blood-fed mosquitoes, as its mRNA based primers are designed to detect L3 specific larvae, so could therefore give an estimation on vector infectivity rates and a direct measure of transmission potential [54].

### MMRR study

This study was conducted to determine the flight range, survival and dispersal of adult *Cx*. *quinquefasciatus* and hence set spatial resolution in a gridded MX system. In this densely populated urban area, the median flight range was 35m, the majority (90%) dispersed within 50m, and the maximum flight range was 85m from the CRP. Of 9644 (7235 female) marked mosquitoes, a total of 10 (8 female, 2 male), or 0.103%, were recaptured. Although this is shorter than other distances reported in the scant literature available on the flight range of *Cx*. *quinquefasciatus*, the most likely explanation is that the richness in host/breeding site availability provided in the urban environment means that a female mosquito does not need to fly far to find blood for egg development or water for oviposition.

Although results from experiments with low or zero recapture rates may be less likely to be published, a recent review of published studies indicates variation in recapture rates between zero and 14% [72]. The recapture rates found in the current study are low but are within the range of MMRR studies for *Cx*. *quinquefasciatus*, which tends to have lower recapture rates compared with other mosquito species [36]. While other methods such as sticky ovitraps could have been used, the requirement of preserving filarial RNA meant that field teams would have had to collect sticky tapes daily or more frequently due to the intense heat and potential predators in the field site; these issues rendered such tools impossible for use.

Aspiration took place within houses and in the peri-domestic area, so it is possible that study teams may have missed marked mosquitoes that did not travel indoors but remained in the narrow pathways of the study site. The low recapture rate may also relate to the marking procedure and its effects. First, marked mosquitoes may have had a lower survival rate compared to wild mosquitoes. Although the effect of the marking was found to be small in the pilot experiment, mosquitoes may still have been harmed during the procedure or transport towards the field site. Second, the color of the mosquitoes removes advantages of their natural camouflage and is likely to make them more vulnerable to predation. Additionally, it is possible that recapture rates may have been higher if collection methods not utilized in the present study, e.g., BG Sentinel traps, were used.

In this study, all mosquitoes were recaptured within a period of four days. This is in concordance with the literature, where recapture success decreases after approximately four to six days [42, 52, 73]. Anecdotally, members of the community reported seeing or killing colored mosquitoes. In particular, the owner of the house on whose property the CRP was located repeatedly reported seeing colored mosquitoes inside the house up to five days after release but these mosquitoes were not recaptured by study teams. One household member of a participating house accidentally killed a magenta-colored mosquito, saved it, and presented it to study teams as evidence (and with an apology): it was a blood fed female mosquito from R3 that had travelled 23.7m before it was killed, reportedly 10–12 hours after its release.

Fewer *Ae*. *aegypti* were collected than *Cx*. *quinquefasciatus*, which is unsurprising given the collection method and deployment schedule. As this study was conducted to design a MX system for LF, the MMRR was primarily interested in *Cx*. *quinquefasciatus* flight distance and survival. Battery-powered aspirators were chosen because they preferentially collect post-blood meal resting females and aspiration occurred from 9am– 11:30am each day in order to coincide with resting *Cx*. *quinquefasciatus*. Adjusting the aspiration schedule towards later in the day would have likely resulted in collecting more *Ae*. *aegypti* mosquitoes.

The furthest recorded distance travelled was 85.4m from the CRP, on day three and at the outer limit of the study area. Hence mosquitoes may have also dispersed beyond the study area. This is difficult to confirm, although *Cx*. *quinquefasciatus* has been reported to travel over 15km (810–1680m) from a release site in other studies [36, 41, 43, 53, 74, 75]. However, the need to migrate long distances seems relatively low in this study area, given the availability of human blood meals in the densely populated urban setting. On the other hand, the relatively small size of the study area may have biased the observed MDT downwards [36].

Although the sample size of recaptured marked mosquitoes was insufficient to perform statistical analysis, most mosquitoes appeared to travel upwind, despite the relatively high wind speeds recorded over the study period. Reisen et al. suggested that *Cx*. *quinquefasciatus* may travel towards areas with vegetation to seek protection against the wind, although Schreiber et al. found they dispersed mainly downwind regardless of land cover [41, 73].

This study required high participation rates from the community, requesting access to every room in participants’ house on a daily basis over four weeks. One house refused access to the field team after the first week and access to other houses was denied on an incidental basis. Reported reasons for refusal were inconvenience caused by the procedure, having visitors and the conception that mosquitoes would return the next day.

### Molecular screening of mosquitoes and immunological screening of study area residents

This study aimed to develop a MX system for the RMR, with the primary interest being in ideal collection method and ascertaining the limits of mosquito dispersion. Unfortunately, the expense of field collection resulted in a limited ability to conduct molecular screening of mosquito samples for *W*. *bancrofti* infection in this current study. Thus, researchers decided to screen a proportion of mosquitoes from each area, and biobank the rest with the hopes of securing future funding for further analysis.

The absence of *W*. *bancrofti* infection in *Cx*. *quinquefasciatus* mosquitoes and the absence of antigen against *W*. *bancrofti* in humans in this small study area does not prove the absence of LF transmission in the RMR. As current infection rates in the active foci are estimated to range between 0.6 and 2%, due to repeated rounds of MDA, much larger sample sizes (>20,000 mosquitoes) would be required to detect *W*. *bancrofti* [9, 28]. Mosquitoes collected for MX of LF are potentially useful for monitoring of other infections, in particular arboviruses such as dengue and Zika, although only if the necessary storage and processing protocols are followed to prevent RNA degradation. Even ignoring the likely clustering of infection, the upper 95% confidence limit for the zero positive tests out of 110 is a prevalence of 3.4% [76].

### Comparison with other MX programs

Among over 10 studies reporting MX program results, the majority have originated from the African (AMRO), Southeast Asian (SEARO), and Eastern Mediterranean (EMRO) Regions of the WHO. This is only the second study to report MX results, however preliminary, from the Pan American (PAHO) region. Moreover, only one MX program has evaluated aspirators in collecting *Cx*. *quinquefasciatus* for *W*. *bancrofti* detection. In contrast to several other studies, this study found overwhelming evidence that large, handheld battery-powered aspirators are extremely effective tools for collection of adult *Ae*. *aegypti* as well as *Cx*. *quinquefasciatus* irrespective of physiological status with the exception of unfed females [60, 77–80]. It should be emphasized that the handheld aspirators used in this study are significantly larger than the handheld, backpack, or mouth aspirators that have been used in other sites (Appendix II).

### Recommendations

This research identifies a role for battery-powered aspiration for MX, having demonstrated that they are extremely effective for collecting not only *Cx*. *quinquefasciatus* but also *Ae*. *aegypti* adult females in this densely populated urban setting. This demonstrates that MX may be promising and feasible where there is the possibility of an integrated LF and arbovirus surveillance program. Although few in number, the recaptured mosquitoes suggest a suitable grid size for MX sampling may be 75 x 75m or slightly larger, based on 90% of mosquitoes dispersing at least 50m and at least one up to 85m. It is possible that in less densely populated or built up urban areas a slightly larger grid (e.g., 100m x 100m) may suffice.

This research team recommends prioritizing considerations of spatial scale and transmission dynamics, including underlying human infection prevalence, when designing grid-based MX systems. MX may seem to require substantial up-front investment in monitoring mosquito populations, especially when human health data (e.g., physician confirmed disease or lab confirmed infection) may already be available. However, with correct design and sufficient time for deployment, mosquito-based MX has the potential to enhance current LF surveillance systems (as well as potentially aid in the early warning of new and cyclical infections such as arboviruses). In settings like the RMR—where, in addition to LF, microcephaly, Zika virus, dengue virus, and chikungunya virus have caused enormous strain on public health resources in recent years—such enhanced disease surveillance systems could be very helpful for planning the allocation of public health resources.

## Supporting information

S1 Appendix Handheld aspirator, CDC light trap, and field deployment. Figure S1. [A] Handheld Aspirator Used in CMC and MMRR; [B] CDC Light Trap Used in CMC; [C] Field Deployment of Handheld Aspirator.
(DOCX)

S2 Appendix Pre-field laboratory marking experiments. (DOCX)

S3 Appendix Screening of adult *C*. *quinquefasciatus* for *W*. *bancrofti*. (DOCX)
