# Supplementary material for: Development of an urban molecular xenomonitoring system for lymphatic filariasis in the Recife Metropolitan Region, Brazil
Source: PLoS Negl Trop Dis. 2018 Oct 16;12(10):e0006816. doi: 10.1371/journal.pntd.0006816 (PMC6203399; doi:10.1371/journal.pntd.0006816)
Supplement: S1 Appendix — Figure S1. [A] Handheld Aspirator Used in CMC and MMRR; [B] CDC Light Trap Used in CMC; [C] Field Deployment of Handheld Aspirator. (DOCX) [file pntd.0006816.s001.docx]

**S1 Appendix. Handheld Aspirator, CDC Light Trap, and Field Deployment**

| 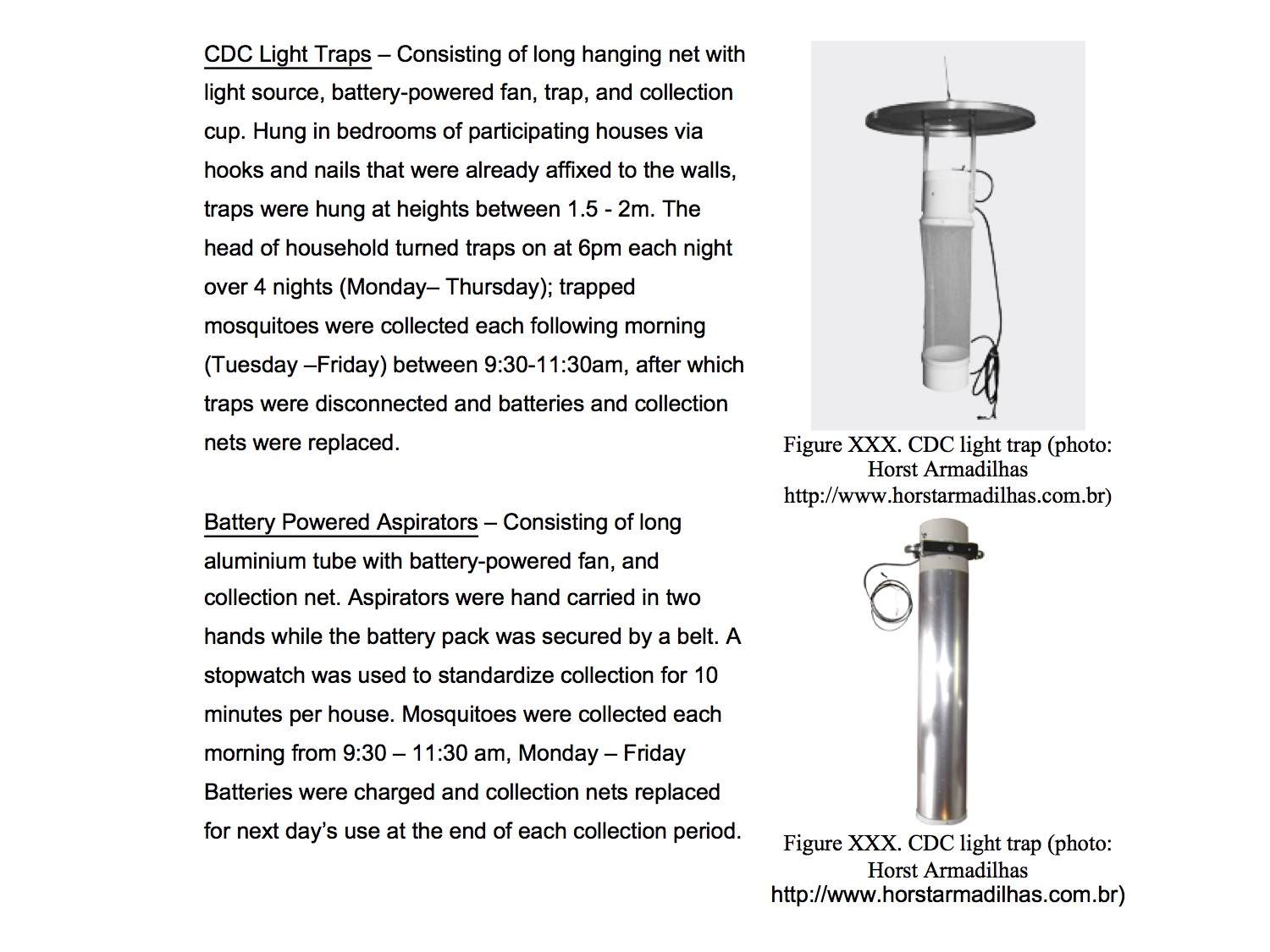 |  | 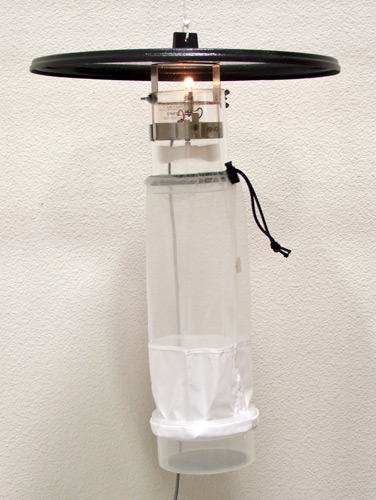 |
| --- | --- | --- |
| **[A] Handheld Aspirator:** Used in CMC and MMRR studies. Plastic base with handles and 12-V battery-powered fan at the top and a long, aluminum body and collection net at the bottom [Photo: www.horstarmadilhas.com.br]. |  | **[B] CDC Light Trap:** Used in CMC study only. Plastic hanging base with light source, battery- powered fan, and trap at the top and a collection net at the bottom [Photo: https://www.bioquip.com]. |
| **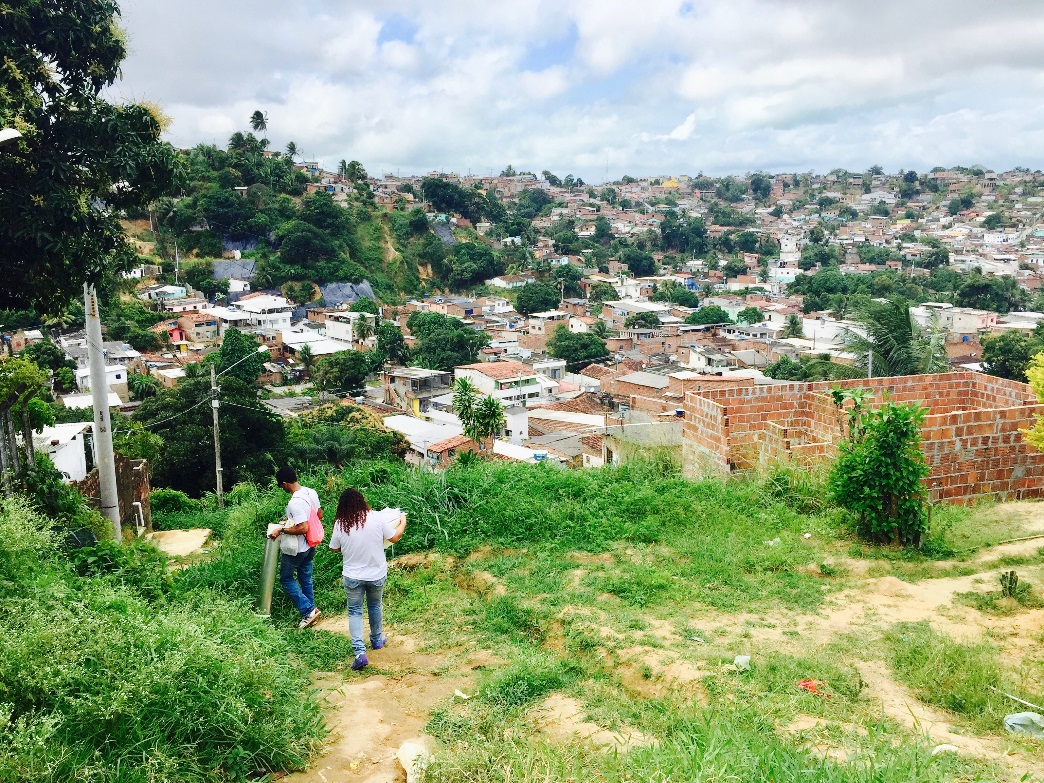** | | |
| **[C] Mosquito Collection in the Field:** Study area and handheld aspirator size  relative to field workers. [Photo: Anita Ramesh, LSHTM and IAM/FIOCRUZ] | | |
| **Fig. A1. [A] Handheld Aspirator (CMC and MMRR); [B] CDC Light Trap (CMC); [C] Field Deployment of Handheld Aspirator.** | | |
