# Supplementary material for: Development of an urban molecular xenomonitoring system for lymphatic filariasis in the Recife Metropolitan Region, Brazil
Source: PLoS Negl Trop Dis. 2018 Oct 16;12(10):e0006816. doi: 10.1371/journal.pntd.0006816 (PMC6203399; doi:10.1371/journal.pntd.0006816)
Supplement: S2 Appendix — (DOCX) [file pntd.0006816.s002.docx]

**S2 Appendix. Pre-Field Laboratory Marking Experiments**

**LSHTM, London, UK [3 weeks, May 2015]:** Laboratory experiments used a *Cx. quinquefasciatus* colony (insecticide susceptible, 45th generation) collected by the Tropical Pesticide Research Institute in Tanzania and held at the LSHTM Insectary since 2010. Adult mosquitoes were fed on filter paper covering a cotton ball soaked in 10% glucose solution before and after marking. Mosquitoes were kept at 27°C with a 12:12 hour photocycle; 60-70% humidity was maintained via plastic covering on cages.

Two age groups (one and eight days old) of mosquitoes were used; for each, two colors were tested to yield four colored groups plus two control groups. The following colors were tested: Red3, Blue, Magenta and Yellow (see A2, next page). For each age group, 25 females were mouth aspirated into a 180ml paper cup. Males were counted when incidentally aspirated. The cup was then placed in a freezer for one minute with recorded temperatures between -12°C and -21.5°C. A 5ml syringe and a 0.6 x 25mm needle was filled up to 0.5 ml with fluorescent powder (Sterling Color, 850 Series, http://www.sterling-colour.co.uk). A dust cloud was created inside the cup by placing the needle at a 90° angle through gauze, blowing the powder in. Mosquitoes were gently moved onto a Petri dish, then placed in cages. Mosquito mortality was recorded at 24-hour intervals over six days, starting from the moment all mosquitoes regained mobility after the marking procedure.

**IAM/FIOCRUZ, Recife, Brazil [1 week, July 2015]:** Laboratory experiments used a *Cx. quinquefasciatus* colony (CqsLab, 45th generation) from the IAM/FIOCRUZ Insectary. Larvae were reared in plastic containers, fed with cat food (Friskies®, fish flavored), and kept between 24.4°C and 27.6°C, with a 12:12 hour photocycle; 63% - 80% humidity was maintained. Adult mosquitoes were held in wire mesh cages, fed on a 10% glucose solution, kept at temperatures between 24.9 and 26.9°C; 66%-81% humidity was maintained via plastic covering on cages. Minor adjustments were made to the methods above based on local availability of materials including (i) using a 0.7mm x 25mm needle instead of a 0.6mm x 25mm needle; and (ii) using plastic 140 ml cups instead of paper 180 ml cups.

|  | **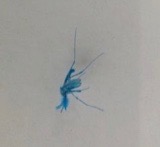** | **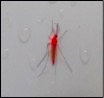** |
| --- | --- | --- |
|  | **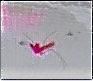** | **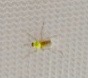** |
| **[A] Fluorescent Dust:** Sterling Color, 850 Series, http://www.sterling-colour.co.uk [Photo: Remy Hoek Spaans] | **[B] Marked Mosquitoes:** Clockwise From Top Left: Blue, Red3, Yellow, Magenta [Photo: Remy Hoek Spaans] | |
| **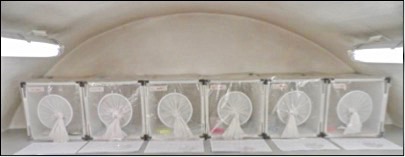** | | |
| **[A] Marked Mosquitoes in Cages**: LSHTM Insectary, May 2015 [Photo: Remy Hoek Spaans] | | |
| **Fig. A2. [A] Fluorescent Dust; [B] Marked Mosquitoes; [C] Marked Mosquitoes in Cages, LSHTM Insectary, May 2015.** | | |
