# Supplementary material for: Development of an urban molecular xenomonitoring system for lymphatic filariasis in the Recife Metropolitan Region, Brazil
Source: PLoS Negl Trop Dis. 2018 Oct 16;12(10):e0006816. doi: 10.1371/journal.pntd.0006816 (PMC6203399; doi:10.1371/journal.pntd.0006816)
Supplement: S3 Appendix — (DOCX) [file pntd.0006816.s003.docx]

**S3 Appendix. Screening adult *Cx. quinquefasciatus* for *W. bancrofti***

**RNA Extraction**: Female *Cx. quinquefasciatus* mosquitoes from each house were pooled in groups of up to 10 and homogenized for 2 min in 150μl of Invitrogen Ultrapure water using a hand-held battery-powered grinder (Fisherbrand Disposable Pestle System). An additional 200μl of water was added before centrifugation at 4000rpm for 20 min at 4°C using an eppendorf Centrifuge 5804R. 100μl of the supernatant was added to 200μl of TRIzol (Ambion) solution, vortexed for 10 sec and left at room temperature for 5 min. 100μl of chloroform was added and each tube was shaken for 15 sec before being left at room temperature for 3 min. Centrifugation at 12,000rpm for 15 min at 4°C resulted in 200μl of clear aqueous phase which was added to 250μl of isopropanol to precipitate RNA. Samples were inverted 10 times and incubated for 10 min at room temperature prior to centrifugation at 12,000rpm for 10 min at 4°C. Supernatant was discarded and RNA pellets were re-suspended in 300μl of 75% ethanol followed by centrifugation for a further 5 min at 12,000rpm and 4°C. Supernatant was removed and RNA pellets were placed at 37°C for 20 min. 30μl of Invitrogen Ultrapure water was added to re-suspend RNA and then briefly vortexed. Samples were stored at -80°C to preserve RNA prior to reverse transcription.

**Reverse Transcription**: RNA samples were reverse transcribed using a QIAGEN QuantiTect reverse transcription kit according to manufacturer’s instructions. Briefly, genomic DNA was removed by adding 2µl gDNA Wipeout Buffer (7x) to 12µl template RNA followed by incubation in a thermal cycler (GeneAmp PCR System 9700, Applied Biosystems) at 42˚C for 2 min, prior to immediately placing the reactions on ice. 6µl of reverse transcription master mix was then added to each sample, comprising 1µl RT Primer Mix, 4µl Quantiscript RT Buffer (5x) and 1µl Quantiscript Reverse Transcriptase per sample. All reactions were prepared on ice. The final reactions were placed in the thermal cycler at 42˚C for 30 min, followed by 95˚C for 3 min before a hold at 4˚C.

**Real-Time PCR (RT-PCR):** In order to confirm successful generation of cDNA from mosquito samples, a *Cx. quinquefasciatus* host SYBR green real time PCR was designed targeting the S7 ribosomal protein (S7) mRNA gene (GenBank Accession # AF272670.1). Primers used were *Cx. quinquefasciatus* FOR: 5’ -AAGGTCGACACCTTCACGTC-3’ and *Cx. quinquefasciatus* REV: 5’-GCGCCGCGAATAGTTTACAG-3’ and the 95 bp target sequence (5’AAGGTCGACACCTTCACGTCGGTGTACAAGAAGCTGACCGGACGCGACGTCACGTTCGAGTTCCCGGAACCCTACCTGTAAACTATTCGCGGCGC-3’) was used to generate a synthetic standard to use as a positive control. PCR reactions were prepared to a total volume of 25µl, containing 12.5µl of 2x QIAGEN QuantiTect Sybr Green Master Mix, 0.3µM final concentrations of each primer, 8.5µl of nuclease-free water and 2µl of template cDNA. Prepared reactions were run on an Applied Biosystems 7500 Fast Real-Time PCR system for 15 min at 95˚C followed by 40 cycles of 94˚C for 15 sec, 55˚C for 30 sec, 72˚C for 30 sec, and a final melt curve analysis as produced by the 7500 software (95˚C for 15 sec, 60˚C for 1 min, continuous detection during ramping to 95˚C for 30 sec, followed by a final 60˚C for 15 sec). Generation of amplified product in each reaction was monitored in real time through acquisition of SYBR green fluorescence readings during the annealing step of each cycle using the SYBR filter and the dissociation of generated products was monitored during the melt curve segment using continuous detection between the second and third steps, using the same SYBR filter.

*W. bancrofti* detection was undertaken using a Taqman real time assay targeting the constitutively expressed *tph-1* gene (GenBank Accession # CD374712.1) for detection of ‘any-stage’ parasites (Laney et al., 2010). Primers used were WuchALL FOR: 5’-GACCGATTTAAACAGTTGCAGTTC-3’ & WuchALL REV: 5’-CTACTACAGCTACTTGTCCCTCACCTT-3’ with a hydrolysis probe (5’-6-FAM-ATCGGTGAGCGTATGGCCGAAGG-TAMRA^™^-3’). The 79 bp target sequence of the *tph-1* gene (5’GACCGATTTAAACAGTTGCAGTTCTTCATCGGTGAGCGTATGGCCGAAGGTCAAGGTGAGGGACAAGTAGCTGTAGTAG-3’) was used to generate a synthetic standard to use as a positive control. PCR reactions were prepared using 10µl of 2x Promega GoTaq Probe qPCR Master Mix, a final concentration of 0.2µM of each primer, 0.1 µM probe, 5µl of nuclease free water and 2µl template cDNA to a final reaction volume of 20µl. 2µl CXR passive reference dye added to each 1ml GoTaq Probe qPCR Master Mix tube on first use. Prepared reactions were run on an Applied Biosystems 7500 Fast Real-Time PCR System for 2 min at 95˚C, followed by 40 cycles of 95°C for 15 sec and 60°C for 1 min. The increase in FAM fluorescence was monitored in real time by acquisition during the combined annealing/extension step of each cycle using the FAM filter. ROX was used as the passive reference dye. Synthetic standard positive controls (a minimum of 3, 10-fold dilutions per run), in addition to no template controls (NTCs) were included on each qPCR run. PCR results were analyzed using the 7500 Fast Software v2.0.6 and the inter-assay Cq values produced by the synthetic standard positive controls were comparable across runs.

**REFERENCES:**

Laney, S. J., et al. (2010). "Detection of Wuchereria bancrofti L3 larvae in mosquitoes: a reverse transcriptase PCR assay evaluating infection and infectivity." PLoS Negl Trop Dis **4**(2): e602.
